# Assessing Cellular Responses in Sepsis^☆^

**DOI:** 10.1016/j.ebiom.2014.10.017

**Published:** 2014-10-28

**Authors:** Jean-Louis Vincent

**Affiliations:** Department of Intensive Care, Erasme University hospital, Université Libre de Bruxelles, Brussels, Belgium

Sepsis – defined as a dysregulated immunological response to infection associated with some degree of associated organ dysfunction (Vincent et al., 2013) – is a common problem and still complicated by unacceptably high mortality rates. Infection is treated with antibiotics, and source control when needed, but there is no effective, specific treatment for sepsis. The only drug that has ever been marketed for this purpose, drotrecogin alfa (activated), was probably efficacious (Vincent, 2012), but was withdrawn, essentially because we could not identify which patients would actually benefit from it. This has been a key problem in many sepsis trials. Indeed, over the years, sepsis has been considered primarily as a *pro*-inflammatory response, so we have focused on controlling pro-inflammatory mediators, including cytokines, such as tumour necrosis factor (TNF) and interleukin-1 (IL-1). However, there is also an *anti*-inflammatory response, which occurs simultaneously (Hotchkiss et al., 2013), and the initial greater pro-inflammatory effect is rapidly followed by a state of immunosuppression (sometimes called “immunoparalysis”). Giving an anti-TNF antibody to patients who are already predominantly immunosuppressed is clearly not desirable and may even be harmful. Similarly, giving the *pro*-inflammatory growth factor, granulocyte colony-stimulating factor (G-CSF), to all septic patients was not associated with improved outcomes (Stephens et al., 2008).

It is clear that sepsis therapies need to be adapted to the individual patient rather than directed at a general diagnosis of ‘sepsis’. The study by Pena et al. (in press) in this issue of *EBioMedicine* represents an important step in this direction. These investigators studied cellular gene expression in peripheral blood mononuclear cells (PBMCs) from septic patients. Using well established gene test approaches, they documented a clear and consistent endotoxin tolerance (also called cellular reprogramming) signature in a series of almost 600 septic patients from different cohorts. The group of investigators is reputed in this field and the results are convincing.

Importantly, cellular reprogramming is not limited to Gram-negative infections and blood endotoxin levels are not increased only in the presence of Gram-negative organisms (Marshall et al., 2004, Buckley et al., 2006). The authors, therefore, proposed that cellular reprogramming could be used as a diagnostic test for sepsis. This is not straightforward conceptually though, because diagnosing sepsis by its induced immunosuppression is like defining a problem by its consequences, and such a test is perhaps applied too late to be of use in facilitating early patient management. Cellular reprogramming may be more helpful to estimate the risk of nosocomial infection than for sepsis diagnosis (Conway Morris et al., 2013), and future studies should evaluate these changes prospectively in patients who are not infected at the time of hospital admission.

The study (Pena et al., in press) has several limitations. Firstly, the authors struggle somewhat with terms and concepts and oscillate between “infection” (a microbiological process typically recognised by the presence of some host response including fever and altered white blood count) and “sepsis” (a more severe form, typically characterised by some degree of organ dysfunction). This is not surprising, because they used the old 1991 criteria for sepsis, in which an infection with fever and increased WBC was indeed called sepsis. Nevertheless, the observation that the sicker the patient, the more likely he/she was to have endotoxin tolerance, suggests that this test may indeed represent a marker of sepsis rather than simply infection. Secondly, few clinical data are provided for the patients studied. Clearly, the investigators started from the blood samples they had, rather than from the clinical scenarios. Moreover, I am always puzzled when I see patients classified in a dichotomised fashion into septic and non-septic, when in clinical practice there are some patients in a “grey zone”, for whom we never know, even at the end of the hospital stay, whether or not there was truly an infection. A typical scenario is the critically ill patient receiving mechanical ventilation who develops a febrile episode associated with a vague chest infiltrate on X-ray, which could correspond to pneumonia but could equally represent some pulmonary oedema or even atelectasis.

Nevertheless, despite these limitations, these data are novel and important, as they confirm the common and early occurrence of immunosuppression following the onset of infection and cellular reprogramming represents a first step in the characterisation of cellular responses in sepsis. Importantly, the definition of “early” sepsis remains difficult. Although more than 80% of the patients in this study were enrolled in the emergency room (ER), suggesting that they were in the early phase of disease, some patients may have been infected some time before reaching the ER; in most patients it is difficult to know when the pathologic process actually began, i.e., when the ‘sepsis clock’ started.

Better characterisation of these responses in individual patients with sepsis will help open the door to immunomodulating strategies, both for clinical trial purposes in allowing better identification of appropriate patient cohorts, but also at a clinical practice level, allowing a more customised approach to therapy with administration of an anti-inflammatory intervention in a pro-inflammatory phase, and an immunostimulating therapy, for example, administration of a cytokine, including IL-7, IL-15, and granulocyte macrophage colony-stimulating factor (GM-CSF), or co-inhibitory molecule blockade, such as anti-programmed cell death receptor-1 (anti-PD-1) and anti-B and T lymphocyte attenuator (Chang et al., 2014, Sherwood and Hotchkiss, 2013), in immunosuppressed phases. Analysis of the individual genetic signature of endotoxin tolerance may thus provide important and clinically relevant information.
